# Cannabinoids Reduce Melanoma Cell Viability and Do Not Interfere with Commonly Used Targeted Therapy in Metastatic Melanoma In Vivo and In Vitro

**DOI:** 10.3390/biology12050706

**Published:** 2023-05-12

**Authors:** Georg Richtig, Melanie Kienzl, Sonja Rittchen, David Roula, Jürgen Eberle, Zina Sarif, Martin Pichler, Gerald Hoefler, Akos Heinemann

**Affiliations:** 1 Otto-Loewi Research Center for Vascular Biology, Immunology and Inflammation, Division of Pharmacology, Medical University of Graz, 8010 Graz, Austria; melanie.kienzl@medunigraz.at (M.K.); sonja.rittchen@medunigraz.at (S.R.);; 2 Division of Oncology, Department of Internal Medicine, Medical University of Graz, 8036 Graz, Austria; martin.pichler@medunigraz.at; 3 BioTechMed-Graz, 8010 Graz, Austria; 4 Department of Dermatology, Venereology and Allergology, Skin Cancer Center Charité, Charité-Universitätsmedizin Berlin (University Medical Center Charité), 10117 Berlin, Germany; juergen.eberle@charite.de (J.E.);; 5 Diagnostic and Research Institute of Pathology, Medical University of Graz, 8036 Graz, Austria; gerald.hoefler@medunigraz.at

**Keywords:** cannabinoid, melanoma, pharmacology, therapy

## Abstract

**Simple Summary:**

Cannabinoids are mainly used for recreational purposes but find their way into oncology due to ongoing legalization efforts and anti-cancerous hints in the scientific literature. The goal of this study was to elucidate the mode of action of a clinically used cannabis medication in metastatic melanoma as well as its clinical value in combination with targeted therapy. By cell viability and apoptosis assays, we could demonstrate that cannabinoids mediate their apoptotic effect in a caspase-mediated fashion by disturbing mitochondrial integrity. With in vivo experiments, we could demonstrate that clinically used cannabinoid medication does not interfere with the commonly used anti-cancerous drug trametinib. Our results suggest that cannabinoids are effective in metastatic melanoma and pave the way for further clinical trials.

**Abstract:**

**Background:** Cannabinoids are mainly used for recreational purposes, but also made their way into oncology, since these substances can be taken to increase appetite in tumour cachexia. Since there are some hints in the literature that cannabinoids might have some anti-cancerous effects, the aim of this study was to study if and how cannabinoids mediate pro-apoptotic effects in metastatic melanoma in vivo and in vitro and its value besides conventional targeted therapy in vivo. **Methods:** Several melanoma cell lines were treated with different concentrations of cannabinoids, and anti-cancerous efficacy was assessed by proliferation and apoptosis assays. Subsequent pathway analysis was performed using apoptosis, proliferation, flow cytometry and confocal microscopy data. The efficacy of cannabinoids in combination with trametinib was studied in NSG mice in vivo. **Results:** Cannabinoids reduced cell viability in multiple melanoma cell lines in a dose-dependent way. The effect was mediated by CB1, TRPV1 and PPARα receptors, whereby pharmacological blockade of all three receptors protected from cannabinoid-induced apoptosis. Cannabinoids initiated apoptosis by mitochondrial cytochrome c release with consecutive activation of different caspases. Essentially, cannabinoids significantly decreased tumour growth in vivo and were as potent as the MEK inhibitor trametinib. **Conclusions:** We could demonstrate that cannabinoids reduce cell viability in several melanoma cell lines, initiate apoptosis via the intrinsic apoptotic pathway by cytochrome c release and caspase activation and do not interfere with commonly used targeted therapy.

## 1. Introduction

Melanoma is the deadliest form of skin cancer, and the therapeutical landscape has broadened over the last years resulting in significant survival benefits [1]. Currently, two major treatment strategies have evolved in malignant melanoma patients: firstly, targeted therapy in the form of B-Raf proto-oncogene, serine/threonine kinase (BRAF) and mitogen-activated protein kinase kinase (MAP2K; MEK) inhibitors in BRAF mutated (*BRAF^mt^*) melanoma and, secondly, immunotherapy. One of the major advantages of targeted therapy in *BRAF^mt^* melanoma is the fact that patients have a high response rate and a rapid onset of action including clinical signs of tumour burden relief. However, one major disadvantage is that patients are rarely completely cured by this type of treatment; therefore, they often rely on additional treatment options offered by ‘alternative medicine’ [2]. Green tea is among the most commonly used natural products for cancer; however, cannabis also has a long history as a supplemental therapy [3,4]. Cannabis is primarily used for ‘recreational’ purposes but has also been proposed to have anti-tumourous and/or anti-inflammatory effects by the scientific literature and other sources [5]. It is important to note that cannabis is not a homogenous substance but rather composed of different chemical entities of at least 60 cannabinoids, whereas some of them have opposing effects [6]. For these reasons, cannabinoids have been investigated as single substances with tetrahydrocannabinol (THC) as the most popular substance due to its psychotropic effects. Up to date, two subtypes of cannabinoid G protein-coupled receptors (GPR), CB_1_ (cloned in 1990) and CB_2_ (cloned in 1993), could be identified [7,8,9]. CB_1_ can signal through G proteins or interact with non-G protein partners such as the adaptor protein FAN [10]. However, cannabinoids are also able to bind to several other receptors. So far, binding has been confirmed for the peroxisome proliferator-activated receptors (PPAR) α and PPARγ, transient receptor potential vanilloid-1 (TRPV1) channels and GPR55 and GPR35 [11].

Since the 1990s there is growing evidence that cannabinoids such as THC as well as endocannabinoids such as 2-arachidonoylglycerol (2-AG) and anandamide and various synthetic cannabinoid receptor agonists may have anti-tumour effects. However, there are also reports suggesting tumour-promoting effects of cannabinoids [12,13].

Here we report that CBD and the combination of CBD and THC can reduce cell viability in different melanoma cell lines. This effect is mediated by activation of the CB1, TRPV1 and PPARα receptors, followed by activation of cytochrome c-mediated cell death. Finally, we demonstrate that cannabinoids have an anti-cancerous effect in vivo and do not interfere with a clinically approved MEK1/2 inhibitor in metastatic melanoma xenograft tumours.

## 2. Materials and Methods

### 2.1. Chemicals and Antibodies

All drugs were purchased from Tocris (Abingdon, UK), except Z-VAD-FMK (Selleckchem, Houston, TX, USA) and THC (Gatt-Koller, Absam, Austria). All antibodies have been ordered from Cell Signaling Technology Europe (Frankfurt am Main, Germany) except Goat anti-Mouse-IgG, Alexa Fluor 488 (Thermo Scientific, Vienna, Austria) and Anti-Cannabinoid Receptor I (Abcam, Cambridge, UK) (Detailed in Supplementary Table S1).

### 2.2. Cell Lines

A375, A2058 and SK-Mel-28 cell lines were purchased from LGC Standards GmbH (Wesel, Germany). A375R, UACC-62 and Colo-800 were a generous gift from Anna Obenauf (Research Institute of Molecular Pathology (IMP), Vienna Biocenter (VBC), Vienna, Austria). SBcl2 was a generous gift from Prof. Beate Rinner (Department for Biomedical Research, Medical University of Graz, Graz, Austria). A2058, A375 and SK-Mel-28 cells were maintained at 37 °C in a humidified atmosphere containing 5% CO_2_ in Dulbecco’s Modified Eagle Medium (DMEM) (Thermo Scientific, Vienna, Austria) supplemented with 10% fetal calf serum (FCS) (Thermo Scientific, Vienna, Austria) and 1% (*v*/*v*) penicillin/streptomycin (P/S). UACC-62 and Colo-800 cells were maintained at 37 °C in a humidified atmosphere containing 5% CO_2_ in Roswell Park Memorial Institute (RPMI) 1640 (Thermo Scientific, Vienna, Austria) supplemented with 10% FCS (Thermo Scientific, Vienna, Austria) and 1% (*v*/*v*) P/S. SBCl2 cells were maintained in a similar way except that 2% FCS instead of 10% was used. For all experimental assays, FCS was reduced from 10% to 2% and from 2% to 0.5%, respectively.

### 2.3. Cell Viability Assay

Cells were seeded into 96-well plates at a density of 5000 cells/100 μL/well and grown for 24 h at 37 °C under a humidified atmosphere containing 5% CO_2_ followed by the desired treatment duration. Cell growth was determined using a commercial kit (MTS Assay Kit; Promega Corp., Madison, WI, USA), according to the manufacturer’s instructions. For measurement of cell proliferation, 10 µL of MTS reagent was added into each well, and cells were incubated light-protected at 37 °C for 1 h, then the absorbance was read at 490 nm with a microplate spectrophotometer (xMark Microplate Absorbance Spectrophotometer; Bio-Rad Laboratories GmbH; Vienna; Austria). All experiments were repeated at least three times in duplicates.

### 2.4. Cell Cycle Analysis

After cells had been detached by trypsin treatment, cells were washed twice with PBS, permeabilized with ice-cold 70% EtOH and incubated overnight at 4 °C. Afterwards cells were incubated with propidium iodide (0.05 mg/mL, Sigma-Aldrich, Vienna, Austria) and Ribonuclease I (0.1 mg/mL, Sigma-Aldrich), followed by incubation at 37 °C for 1 h. Stained cells were immediately analysed by flow cytometry on a BD FACScan Flow-Cytometer (Becton Dickenson Heidelberg, Germany). The percentage of cells in each cell cycle phase was analysed with FlowJo software (FlowJo LLC, Ashland, OR, USA).

### 2.5. AnnexinV/PI Co-Staining

A2058 cells were seeded in 24-well plates (50,000/well) and kept in DMEM containing 2% FCS and 1% P/S. Cells were treated—if indicated—with an antagonist (AM251, 9 µM; AMG9810, 50 µM; GW6471, 15 µM; BafA1, 100 nM; Z-VAD, 100 µM, trametinib, 30 nM or vemurafenib, 1 µM) at 20 min prior to treatment with cannabinoids (C + T 6 µM; CBD 6 µM; CBD 10 µM; THC 6 µM; THC 10 µM or THC 15 µM) or chloroquine (50 µM)—if not otherwise specified—for 24 h at 37 °C. After trypsinization, cells were washed with PBS, collected via centrifugation and stained with Annexin V and PI for 15 min protected from light at room temperature according to the manufacturer’s protocol (Annexin V-FITC Apoptosis Detection Kit I, BD Pharmingen). Analysis was performed using a BD FACScan Flow-Cytometer (Becton Dickenson Heidelberg, Germany) and FlowJo (FlowJo LLC, Ashland, OR, USA).

### 2.6. Caspase-3/7—Glo Assay

A2058 cells were seeded into 96-well plates (0.5 × 10^5^/per well) and cultured for 24 h in DMEM containing 2% FCS and 1% P/S supplemented with the indicated treatment. In some experiments, cells were pre-treated with antagonists 20 min prior to the treatment with cannabinoids (CBD, THC or C + T). Caspase-3/7 activation was determined using a commercial kit (Caspase-Glo 3/7 Assay; Promega Corp., Madison, WI, USA), according to the manufacturer’s instructions. For the measurement of caspase activation, 50 µL of caspase reagent was added into each well, and cells were incubated light-protected at 37 °C for 1 h; then, the absorbance was read at 490 nm with a microplate spectrophotometer (PerkinElmer Topcount NXT; Waltham, MA, USA).

### 2.7. JC-1 Staining

Cells (5 × 10^4^) were kept in DMEM supplemented with 2% FCS and 1% P/S and incubated with antagonists 20 min prior to the indicated treatment with cannabinoids (CBD, THC or C + T) for the indicated time points and concentrations or for 24 h—if not otherwise mentioned—at 37 °C. The mitochondrial uncoupler Carbonyl cyanide-4-(trifluoromethoxy)phenylhydrazone (FCCP) (1 nM) served as a positive control. After cell detachment, cells were washed twice in PBS and then incubated in JC-1 dye (2 μg/mL, Thermo Fisher Scientific) for 20 min at 37 °C. Flow cytometric measurements were performed using a BD FACScan Flow-Cytometer (Becton Dickenson Heidelberg, Germany) for the detection of mitochondrial depolarization. The intact mitochondrial membrane is reflected by JC-1 monomers that were detected in the FL-1 channel. Depolarization of the mitochondrial membrane led to the formation of orange J-aggregates that were measured in the FL-2 channel [14].

### 2.8. Crystal Violet Assay

For the quantitative determination of cells adhering to the plate after the 24 h treatment with different concentrations of CBD, THC and PPARα, the crystal violet assay was used. Cells were carefully washed after treatment and stained with crystal violet solution (0.05% crystal violet, 1% formaldehyde, 1% methanol in PBS) for 20 min. After careful washing with PBS, plates were dried overnight, methanol was added and reading was performed with a spectrometer (VICTOR Multilabel Plate Reader, PerkinElmer, Waltham, MA, USA) at 560 nm.

### 2.9. Immunohistochemistry

Immunohistochemistry was performed on 4 μm thick formalin-fixed paraffin-embedded (FFPE) tumour sections from every single mouse. Staining was carried out using the Dako Omnis platform (Agilent, Santa Clara, CA, USA). Ki67 (Agilent, Santa Clara, CA, USA), and S100 (Agilent, Santa Clara, CA, USA) staining was carried out according to the manufacturer’s protocol. In summary, pre-treatment was performed using the DAKO OMNIS EnVision FLEX Target Retrieval Solution Low pH solution (Agilent, Santa Clara, CA, USA) for 30 min at 97 °C (Ki67 and S100), followed by blocking using peroxidase from the EnVision FLEX Mini Kit, High pH, (Agilent, Santa Clara, CA, USA) for 3 min. Incubation time for S100 was 12 min at 25 °C and for Ki67 20 min at 25 °C. As a detection system, the ENV FLEX HRP from the EnV FLEX, High pH, (Dako Omnis) (Agilent, Santa Clara, CA) was used. Ki67 and S100 samples were treated for 20 min at 25 °C without any linker with the ENV FLEX HRP detection system. Afterwards, samples were incubated for 5 min at 25 °C with DAB+ substrate chromogen (Agilent, Santa Clara, CA, USA). Counter-staining was performed using hematoxylin (Merck, Darmstadt, Germany) for 1 min at room temperature. Between each step, samples were washed with a wash buffer 20× (Agilent, Santa Clara, CA, USA).

Following immunohistochemical staining, tumour sections were scanned using Pannoramic 1000 (3D Histech, Budapest, Hungary), whereby 5 non-overlapping regions were randomly selected (35× magnification) and extracted for quantification of nuclear Ki67 staining with QuPath Software [15]. Additionally, the S100 expression was evaluated in one randomly chosen image. One image resulted in approximately 2000 cells for quantification. Expression analysis was performed in batch mode, whereby nuclei were detected according to hematoxylin counterstain with the following standard settings: background radius 15 px, Sigma 3 px, minimum area 10 px^2^, maximum area 1000 px^2^ and intensity threshold 0.1. Cell expansion was set at 5 px. For the characterization of Ki67+ cells, only the nuclear score compartment considering DAB OD mean was chosen, whereby threshold 1 was 0.2, threshold 2 was 0.5 and threshold 3 was 1. S100 staining was evaluated using the whole cell as a score compartment (DAB OD mean) with thresholds of 0.1, 0.2 and 0.6. The thresholds were set by a treatment-blinded person after the evaluation of 10 images chosen randomly from the data set.

### 2.10. Cancer Genomics

Data from The Cancer Genome Atlas (TCGA) and the Cancer Cell Line Encyclopedia were accessed through the www.cbioportal.org website on 19 November 2018. The database was searched for mutations in CNR1, PPARα and TRPV1 for the skin cutaneous melanoma (SKCM) cohort (366 patients) and the Cancer Cell Line Encyclopedia (1020 cell lines). Mutational frequencies—depending on data availability—were summarized and plotted using the output provided by the online portal.

### 2.11. In Vivo Experiments

Ten- to twenty-six-week-old male NOD Scid gamma (NSG) mice were a generous gift of Dr. Andreas Reinisch (Medical University of Graz, Graz, Austria) and had originally been obtained from Charles River Laboratories (Wilmington, MA, USA). A2058 cells (1 × 10^6^ cells/mouse) were injected subcutaneously (s.c.) in the area of the right flank.

The mice were bred and kept under specific pathogen-free conditions in the animal facility of the Medical University of Graz (Graz, Austria). All experimental procedures were done according to European Guidelines and were approved by the national animal ethics committee (BMBWF-66.010/0139-V/pot3b/2019). Seven days after the injections, when tumours were palpable in all mice, they were randomized to treatment groups in a blinded fashion (8 mice per group). There were no significant differences regarding age and weight among all four groups. C + T (CBD 10 mg/kg BW and THC 10 mg/kg BW) and trametinib (Selleckchem, Houston, TX, USA) (0.75 mg/kg BW) were dissolved in ethanol and kolliphor (Merck, Darmstadt, Germany) one to one. Before use, drugs were further diluted 1/10 with phosphate-buffered saline (PBS). Subcutaneous injections were given once per day (150 μL/mouse) for 21 days. Control mice were injected with equal amounts of kolliphor/ethanol dissolved in PBS. Treatment-related toxicity was determined by monitoring mouse weight and appearance every day. The tumour size was measured with a caliper, and tumour volume was calculated according to the following equation: *Volume* = 0.5 × (*length* × *width*^2^) [16]. Mice were sacrificed on day 22 after treatment initiation, and tumour samples were collected for further analysis.

### 2.12. Statistical Analysis

GraphPad Prism version 6 (GraphPad Software Inc., San Diego, CA, USA) was used to perform statistical tests. Bar plots and graphs were created with GraphPad or ggplot2. All data are shown as mean + SD for n observations. For three or more groups, one-way or two-way ANOVA followed by Dunnett’s, Tukey or Bonferroni multiple comparison test were used or, for two groups, a Mann–Whitney-U test was used. A two-sided *p* value of <0.05 was considered statistically significant and indicated as * = *p* < 0.05; ** = *p* < 0.01; *** = *p* < 0.001 and **** = *p* < 0.0001.

## 3. Results

### 3.1. Cannabinoids Reduce Cell Viability among a Variety of Melanoma Cell Lines in a Concentration-Dependent Manner

**Figure 1:**
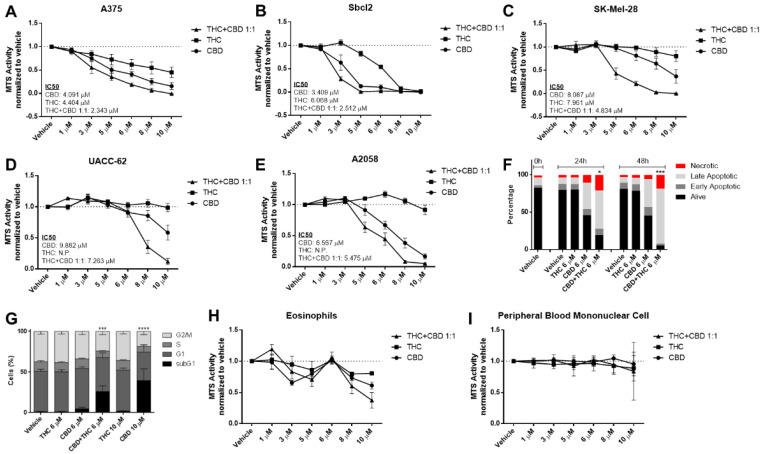
**Cannabinoids decrease cell viability of melanoma cell lines but not in non-cancerous control cells in a concentration-dependent way.** (**A**–**E**) Cell viability levels were assessed by MTS assay of different melanoma cell lines treated with increasing dosage of cannabidiol (CBD), tetrahydrocannabinol (THC) or a combination of both (1:1 ratio) for 24 h. (**F**) A2058 melanoma cells were treated with 6 µM of CBD, 6 µM of THC or a combination of both for 24 and 48 h, followed by AnnexinV/PI staining and flow cytometric analysis. Alive cells were AnnexinV/PI negative, early apoptotic cells were AnnexinV positive but PI negative, late apoptotic cells were AnnexinV/PI double positive and necrotic cells were only PI positive. (**G**) A2058 cells were treated with 6 or 10 µM of CBD or THC or a 6 µM combination of CBD and THC for 24 h. Flow cytometric cell cycle analysis was performed using PI staining. (**H**,**I**) Eosinophils and peripheral blood mononuclear cells were treated with increasing concentrations of CBD, THC or a combination of both (C + T; 1:1 ratio) for 24 h, followed by cell viability assessment by MTS assay. All experiments were performed in duplicates and at least 5 times. Data are presented as mean ± SD; ns = not significant; * = *p* < 0.05; ** = *p* < 0.01; *** = *p* < 0.001 and **** = *p* < 0.0001; IC50 = half maximal inhibitory concentration; N.P. = not possible.

Since melanoma is a highly heterogenous type of tumour with the highest mutational burden among all cancer types [17] and, therefore, comes with a higher probability of primary resistance to any pharmacological therapy, we aimed to investigate the efficacy of cannabinoids in multiple mutationally different melanoma cell lines. The commonly used *BRAF^V600E^* non-metastatic, horizontally growing melanoma cell line A375 showed a concentration-dependent reduction in cell viability when treated with THC or CBD (Figure 1A).

Similarly, the non-metastatic *NRAS^Q61K^* mutated cell line SBcl2 showed a concentration-dependent reduction of viability (Figure 1B). Sativex (GW Pharmaceuticals, UK) is a 1:1 combination of THC and CBD at similar proportions (C + T) and was tested in different cancer types [18,19,20]. In line with these results, we observed that CBD plus THC had an additive effect that was stronger than either drug alone (Figure 1A,B). Some key mutations have been identified that are present in metastatic melanoma and are known to drive metastatic capability [21]. For this reason, we investigated the efficacy of cannabinoids in cell lines with a metastatic phenotype harbouring mutations in the BRAF, TP53 and PTEN genes (summarized in Table S2).

New therapeutic approaches are urgently needed in melanoma due to primary and secondary resistance in *BRAF^mt^*. THC up to 10 μM had no effect on cell viability of BRAF^mt^ TP53^mt^ cell lines SK-Mel-28, UACC-52, A2058 and Colo-800 in contrast to the A375 and SBcl2 cell lines (Figure 1C–E and Supplementary Figure S1A), whereas CBD and CBD plus THC (C + T) reduced cell viability in all cell lines in a concentration-dependent manner.

The metastatic cell line A2058 harbours a *MAP2K1^P124S^* mutation that makes it naturally resistant to BRAF inhibitor therapy [22], which we confirmed with vemurafenib in the MTS assay (Figure S1B). AnnexinV/PI staining confirmed a pro-apoptotic effect of C + T at 24 and 48 h (Figure 1F). Similar effects were seen in cell cycle analysis after 24 h for C + T and for CBD at higher concentrations (Figure 1G and Figure S1C).

It is known that cannabinoids may exhibit anti-inflammatory properties [23]. Conversely, anti-tumourigenic activity by immune cells, or the lack of it, is a critical factor during cancer therapy or cancer development, respectively. Hence, we next aimed to investigate the effect of cannabinoids on immune cell viability. Firstly, it has been shown that eosinophils might play an essential role in the facilitation of immune therapy [24] and cannabinoids reduced cell viability of freshly isolated eosinophils in a concentration-dependent bi-phasic way (Figure 1H). Secondly, since lymphocytes and monocytes also play an important role in tumour control [25,26], we investigated the effect of cannabinoids on peripheral blood mononuclear cells. Notably, there was no effect on cell viability within the given concentration range (Figure 1I). Since there was no effect observed in the tested immune cells (Figure 1H,I) when CBD 6 μM and THC 6 μM were given, we performed all subsequent experiments with a 1:1 combination of this concentration.

### 3.2. Cannabinoids Mediate Their Effects by Activation of CB1, TRPV1 and PPARα Receptors

**Figure 2:**
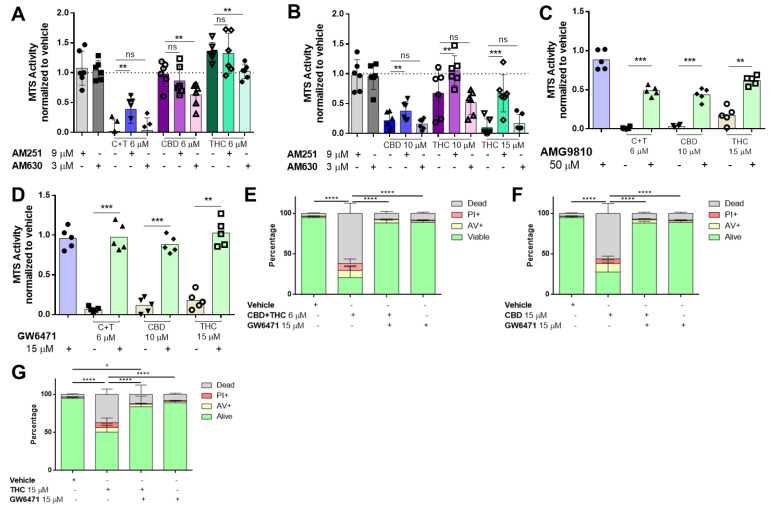
**Cannabinoids mediate their effect through the TRPV1, CB1 and PPARα receptors in melanoma cells.** A2058 melanoma cells were pre-treated with 9 µM of AM251 (CB1 antagonist) or 3 µM of AM630 (CB2 antagonist) prior to treatment with (**A**) 6 µM of CBD, THC or a 1:1 combination of both (CBD 6 µM and THC 6 µM; (C + T)), or (**B**) 10 µM of CBD, THC or 15 µM of THC for 24 h, followed by cell viability assessment using the MTS assay. Cells were treated with (**C**) 50 µM AMG9810 (TRPV1 antagonist) or with (**D**) 15 µM of GW6471 (PPARα antagonist) prior to treatment with 10 µM CBD, 15 µM THC or a 6 µM combination of CBD and THC (1:1), followed by MTS cell viability assessment or by (**E**–**G**) AnnexinV/PI co-staining (Dead: AnnexinV−/PI+, Alive: PI−/AnnexinV−). All experiments were performed in duplicates and at least 5 times. Data are presented as mean ± SD; ns = not significant; * = *p* < 0.05; ** = *p* < 0.01; *** = *p* < 0.001 and **** = *p* < 0.0001.

It has been widely suggested that cannabinoids mediate their effects via several receptors including the family of cannabinoid receptors (CB), transient receptor potential cation channel subfamily V (TRPV) [27] and the peroxisome proliferator-activated receptor (PPAR) family [28]. To identify the receptors involved in cannabinoid signalling, cells were pre-treated with inhibitors against PPARα (GW6471), PPARγ (GW9662), CB1 (AM251), CB2 (AM630) and TRPV1 (AMG9810), followed by stimulation with cannabinoids for 24 h. Pre-treatment with a CB1 receptor antagonist significantly increased viability in A2058 melanoma cells treated with C + T, whereas the CB2 receptor antagonist had no effect (Figure 2A,B and Figure S1D).

In cells that received higher concentrations of CBD and THC, CB1 receptor antagonist pre-treatment also significantly increased viability, whereas CB1 antagonists had more prominent effects on THC than CBD (Figure 2B). However, these effects were not confirmed by AnnexinV/PI staining (Figure S1D). When a potent TRPV1 inhibitor (AMG9810) was used in the MTS assay, we observed that viability was enhanced in C + T (6 μM), CBD (10 μM) and THC (15 μM) treated cells (Figure 2C), and this was also confirmed when AnnexinV/PI staining was used (Figure S1E).

Several studies demonstrated that both PPARα and PPARγ could be involved in cancer apoptosis [28]. To identify whether PPARα or PPARγ were responsible for mediating a pro-apoptotic effect by cannabinoids, we used two specific antagonists (PPARα: GW6471 and PPARγ: GW9662). When the PPARγ antagonist (GW9662) was tested, we did not observe any effect on cell viability (Figure S2A).

However, the PPARα antagonist (GW6471) robustly salvaged cell viability in all conditions investigated (Figure 2D and Figure S2B–E). Importantly, this was confirmed by AnnexinV/PI staining in cells treated with C + T (Figure 2E), high-concentration CBD (Figure 2F) and high-concentration THC (Figure 2G).

Since melanoma is a genetically highly heterogenous disease, we were interested if CNR1, PPARα and TRPV1 are frequently mutated and therefore not accessible for inhibitory therapy. Hence, we performed The Cancer Genome Atlas (TCGA) search. In 366 patients’ cases and the Cancer Cell Line Encyclopedia from Novartis and Broad Institute, which were analysed for mutations in CNR1, TRPV1 and PPARα, up to 3% were mutated (with no mutational hotspot) (Figure S2F,G). This may indicate that targeting the cannabinoid pathway could potentially be a robust pharmacological approach due to the low numbers of mutations within this pathway.

### 3.3. Cannabinoids Impair Mitochondrial Integrity

**Figure 3:**
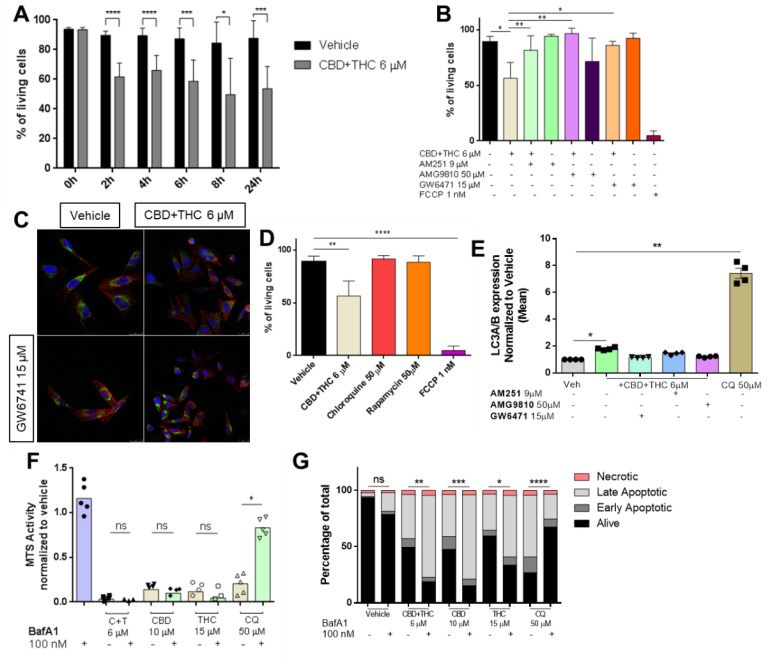
**Cannabinoids impair mitochondrial integrity.** (**A**) Cells were treated with 6 µM of a combination of CBD and THC (ratio 1:1) for 0, 2, 4, 6, 8 and 24 h followed by JC-1 staining and flow cytometric analysis for assessment of mitochondrial integrity. (**B**) Prior to the combined treatment of 6 µM of CBD and THC (ratio 1:1) for 24 h, A2058 cells were treated with 9 µM AM251 (CB1 antagonist), 50 µM AMG9810 (TRPV1 antagonist) or 15 µM GW6471 (PPARα antagonist) followed by JC-1 staining and flow cytometric analysis. An amount of 1 nM of FCCP was used as a positive control. (**C**) Cells were treated with 6 µM of a combination of CBD and THC for 2 h with or without prior treatment with 15 µM of GW6471. After treatment, cells were stained for their nuclei (DAPI staining, blue), cytochrome c (green) and their actin skeleton (phalloidin, red), and representative images were taken. (**D**) Cells were treated with vehicle, 6 µM of CBD and THC, 50 µM of chloroquine, 50 µM of rapamycin or 1 nM of FCCP for 24 h followed by JC-1 staining and flow cytometric analysis. (**E**) Prior to treatment with 6 µM of a combination of CBD and THC for 24 h, A2058 cells were treated with 9 µM of AM251, 50 µM of AMG9810 or 15 µM of GW6471, stained for LC3A/B expression and analysed by flow cytometric. (**F**,**G**) A2058 cells were treated with 100 nM of BafA1 followed by treatment with 6 µM of CBD and THC (C + T), 10 µM CBD, 15 µM THC or 50 µM chloroquine for 24 h followed by cell viability assessment by (**F**) MTS assay or (**G**) AnnexinV/PI co-staining. Data are presented as mean ± SD; ns = not significant; * = *p* < 0.05; ** = *p* < 0.01; *** = *p* < 0.001 and **** = *p* < 0.0001.

Mitochondria play a crucial role in cell metabolism and are often targeted by apoptotic processes [29]. Therefore, we were interested whether cannabinoids can impair mitochondrial membrane potential (ΔΨm) and tested this by flow cytometry using a cationic dye (JC-1), indicating mitochondrial membrane potential changes. Indeed, CBD and C + T impaired mitochondrial integrity after 24 h and C + T and after 48 h of treatment (Figure S3A). For C + T, this impairment started as early as 2 h after initial treatment (Figure 3A) although cell counts remained stable for up to 24 h (Figure S3B).

Next, we were interested in whether mitochondrial depolarization can be reversed by blockade of CB1R, TRPV1 or PPARα prior to treatment with C + T. As anticipated, mitochondrial depolarization was significantly reduced when pre-treatment was performed with a CB1, TRPV1 or PPARα antagonist (Figure 3B and Figure S3C–E).

One further step of proapoptotic mitochondrial activation is the release of cytochrome c [29]. When cells were treated with C + T for 2 h, a reduction of cytochrome c content was observed, which was prevented by pre-treatment with GW6471 (Figure 3C). In the literature, the mode of action of cannabinoids has been associated with autophagy, whereas it has been suggested that mitochondria and autophagy initiation are linked by p53 activation [30,31]. Two substances capable of inducing autophagy are rapamycin and chloroquine [31]. Surprisingly, neither chloroquine nor rapamycin had any significant impact on mitochondrial integrity in contrast to C + T in tested A2058 cells (Figure 3D). To test whether autophagy is involved in cannabinoid-mediated mitochondrial-driven cell death, we performed flow cytometry staining of LC3A/B in cells treated with C + T for 24 h. As suggested in the literature, chloroquine and C + T significantly induced autophagy-related LC3A/B vesicles. Pre-treatment with PPARα antagonist, but not with CB1 or TRPV1 antagonists, significantly abolished this effect (Figure 3E). Next, we were interested if pre-treatment with bafilomycin A1 (BafA1)—a potent autophagy inhibitor—was able to increase viability in cannabinoid-treated cells. In contrast, BafA1 pre-treatment was only capable to increase viability in chloroquine-treated cells but not in cannabinoid-treated cells (Figure 3F). Surprisingly, AnnexinV/PI staining revealed that a blockade of autophagy with BafA1 increased the number of apoptotic cells when combined with high-concentration CBD (10 µM), high-concentration THC (15 µM) or a low-concentration combination of both (C + T 6 µM) (Figure 3G), suggesting that autophagy might not be significantly involved in cannabinoid-induced cell death.

**Figure 4:**
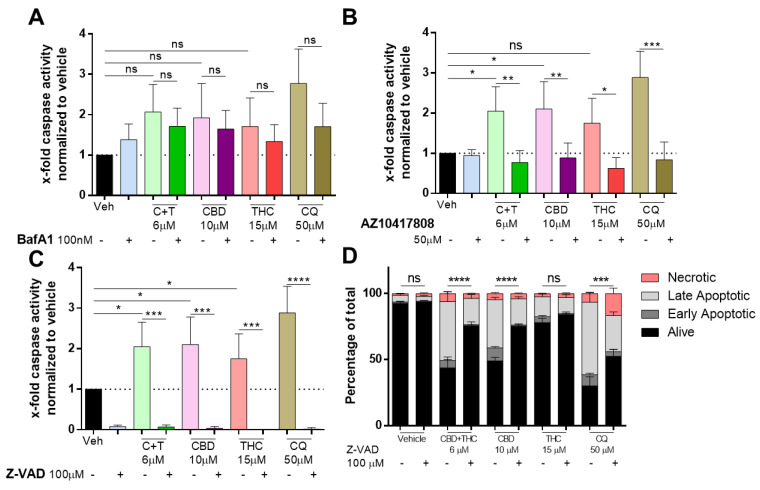
**Cannabinoids mediate their apoptotic effect in a caspase-dependent way.** A2058 cells were pre-treated with (**A**) 100 nM of BafA1, (**B**) 50 µM of AZ10417808 or (**C**,**D**) 100 µM of Z-VAD and afterwards treated with 6 µM of CBD and 6 µM of THC (C + T; 1:1 ratio), 10 µM of CBD, 15 µM of THC or 50 µM of chloroquine (CQ) for 24 h. (**A**–**C**) Caspase activity was assessed by caspase-glo assay, and (**D**) apoptosis was assessed by AnnexinV/PI staining and flow cytometric analysis (Alive: AnnexinV−/PI−; Early Apoptotic: AnnexinV+/PI−; Late Apoptotic: AnnexinV+/PI+ and Necrotic: AnnexinV−/PI+). All experiments have been performed in duplicate five times. Data are presented as mean ± SD; ns = not significant; * = *p* < 0.05; ** = *p* < 0.01; *** = *p* < 0.001 and **** = *p* < 0.0001.

Caspases are widely known as the key modulators of apoptotic cell death and a recent report suggested that BafA1-mediated cell death is independent of caspase activation [32,33]. Pre-treatment with BafA1 followed by cannabinoid or chloroquine treatment for 24 h did not significantly reduce caspase-3/7 activity in A2058 melanoma cells (Figure 4A).

Additionally, we were interested in whether caspase activity induced by cannabinoids can be reversed by the caspase-3 specific inhibitor AZ10417808 [34], and whether caspase activation is induced by CBD or THC treatment. As shown in Figure 4B, AZ10417808 significantly reduced caspase activity not only in cannabinoid-treated cells but also in chloroquine-treated cells. Accordingly, the pan-caspase inhibitor Z-VAD-MFK (ZVAD) prevented caspase activity to non-detectable levels in cells treated with C + T (6 µM), CBD (10 µM), THC (15 µM) and chloroquine (50 µM) (Figure 4C). We assessed whether ZVAD could prevent apoptosis. AnnexinV/PI staining revealed that in cells treated with C + T (6 µM), CBD (10 µM) and chloroquine (50 µM), but not in vehicle and THC (15 µM) treated cells, ZVAD significantly increased the fraction of viable cells (Figure 4D). Finally, we assessed whether other major apoptosis pathways including XIAP/IAP, JNK or mTOR were involved in cannabinoid-mediated apoptosis. However, inhibitors against XIAP/IAP (UC112) (Figure S3F), JNK (BI78D3) (Figure S3G) or mTOR (Rapamycin) (Figure S3H) were not capable of restoring impaired viability in cannabinoid-treated cells. In contrast, pre-treatment with rapamycin (5 µM) further decreased viability when combined with CBD (10 µM) or THC (15 µM) in A2058 cells.

### 3.4. Cannabinoids Do Not Interfere with Commonly Used Targeted Therapy

**Figure 5:**
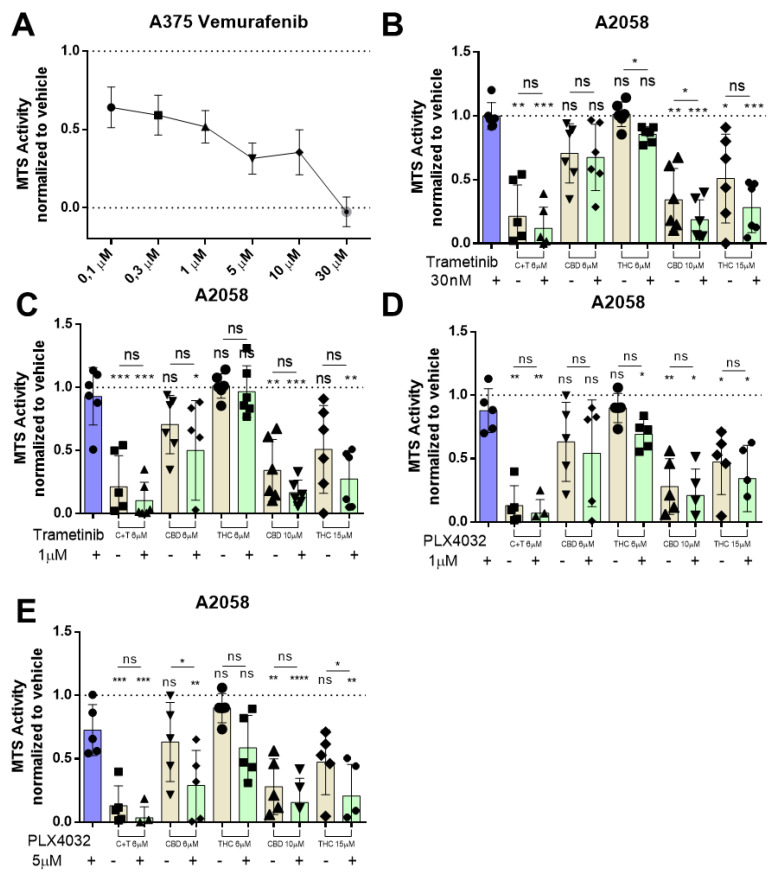
**BRAF and MEK inhibitor therapy has no antagonistic effect on cannabinoid treatment in metastatic melanoma cell lines.** (**A**) A375 cells were treated with increasing dosages (100 nM, 300 nM, 1 µM, 3 µM, 10 µM, 30 µM) of vemurafenib for 24 h, and cell viability was assessed by MTS assay afterwards. A2058 cells were pre-treated with (**B**) 30 nM or (**C**) 1 µM of trametinib or with (**D**) 1 µM or (**E**) 5 µM of PLX4032 (Vemurafenib) followed by treatment with 6 µM of CBD and 6 µM of THC (ratio 1:1; C + T), 6 µM of CBD, 6 µM of THC, 10 µM of CBD or 15 µM of THC. Cell viability was assessed by MTS assay. All experiments have been performed in duplicate five times. Data are presented as mean ± SD; ns = not significant; * = *p* < 0.05; ** = *p* < 0.01; *** = *p* < 0.001 and **** = *p* < 0.0001.

We investigated whether cannabinoids would alter the sensitivity of melanoma to BRAF inhibitor therapy. Thus, we tested the clinically used BRAF inhibitor vemurafenib in A2058 metastatic melanoma cells in comparison to the non-metastatic melanoma cell line A375. Vemurafenib reduced cell viability in a concentration-dependent way in A375 cells (Figure 5A), whereas nearly no reduction was seen in A2058 cells until 30 µM of vemurafenib were used, which is in line with the current literature [35] (Figure S1B).

Moreover, A2058 cells were also insensitive to the MEK inhibitor trametinib up to 10 µM when treated for 24 h (30 nM to 10 µM) (Figure S4A). This raised the question of whether cannabinoids might be able to resensitize the metastatic melanoma cell lines for MEK inhibitor therapy. A2058 cells were pre-treated for 30 min with trametinib followed by different concentrations of THC, CBD or C + T for 24 h. When a low concentration of trametinib was used (30 nM), we could observe a statistically significant reduction in cell viability when combined with THC 6 µM or CBD 10 µM (Figure 5B). When the concentration of trametinib was raised to 1 µM and again combined with cannabinoids, no combination could achieve a significant reduction in viability after 24 h (Figure 5C). Pre-treatment with 1 µM vemurafenib did not significantly reduce A2058 cell viability when combined with different concentrations of cannabinoids (Figure 5D). After pre-treatment with 5 µM vemurafenib, we observed a significant reduction in cell viability when combined with CBD 6 µM and THC 15 µM (Figure 5E), suggesting that cannabinoids might be able to resensitize some melanoma cells for BRAF inhibitor therapy.

### 3.5. Cannabinoids Delay Melanoma Growth In Vivo

**Figure 6:**
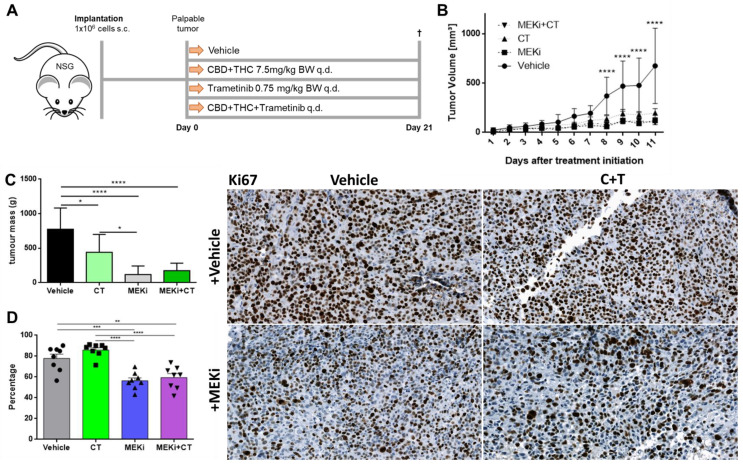
**Cannabinoids and commonly used targeted therapy significantly reduce tumour volume in vivo.** (**A**) Schematic outline of the in vivo procedure. Briefly, 1 × 10^6^ A2058 cells were injected in NOD.Cg-*Prkdc^scid^ Il2rg^tm1Wjl^/SzJ* (NSG) mice and grown until the tumour was palpable but not longer than two weeks. Eight mice were allocated to each group. Group one was treated with vehicle, group two with the combination of CBD and THC in a 1:1 ratio (C + T), group three with the commonly used targeted therapy agent trametinib (MEKi) and group four with all three drugs together (MEKi + C+T). Mice were treated with the allocated treatment (s.c.) every day, sacrificed on day 21 after treatment initiation and tumours were excised for further analysis. (**B**) Tumours were measured every day with a caliper and tumour volume was calculated using the following formula: *Volume* = 0.5 × (*length* × *width*^2^) [16]. (**C**) Ex vivo tumour weight was determined on day 21. (**D**) Tumours were immunohistochemically stained for the proliferation marker Ki67. Data are presented as mean ± SD; ns = not significant; * = *p* < 0.05; ** = *p* < 0.01; *** = *p* < 0.001 and **** = *p* < 0.0001.

Finally, we tested the effects of cannabinoids on melanoma growth in vivo using NOD.Cg-*Prkdc^scid^ Il2rg^tm1Wjl^/SzJ* (NSG) mice. One million A2058 cells were injected into the right lower flank of the mice and treatment was initiated when the tumours were palpable (Figure 6A).

Thereafter, mice were treated every day for 21 days with trametinib (MEKi), a 1:1 combination of CBD and THC or a combination of all. Since all mice came from the 3R program, they differed in age and weight and had to be equally distributed among the groups. There was no significant difference among the groups regarding the age of the mice (Figure S4B), but the MEKi group was heavier than the vehicle and the cannabinoid plus MEKi group (Figure S4C).

Eight days after treatment initialization, all three treatment groups showed a significant reduction in tumour volume (Figure 6B) and in tumour area (Figure S4D) as compared to vehicle only. This effect remained significant until the end of the experiment (day 21). However, there was no statistically significant difference between the single treatment groups (C + T vs. MEKi, MEKi vs. C + T+MEKi, C + T vs. C + T+MEKi). When tumours were excised and ex vivo tumour mass was determined, there was a significant reduction of tumour mass in all treatment groups compared to vehicle (Figure 6C). In addition, MEKi alone was capable of reducing tumour mass more efficiently than C + T. Finally, when excised tumours were stained for the commonly used proliferation marker Ki67, there were significantly reduced numbers of Ki67-positive cells in the groups treated with trametinib (MEKi) alone or in combination with C + T (Figure 6D), whereas the expression of the commonly used immunohistochemistry melanoma marker S100 [36] remained stable across all groups (Figure S4E). Surprisingly, C + T alone did not significantly reduce the number of Ki67-positive cells as compared to vehicle. These in vivo experiments demonstrated that cannabinoids have a strong anti-tumour effect, which, however, differs from that of the MEK inhibitor trametinib.

## 4. Discussion

In this study, we demonstrated that (i) cannabinoids have toxic, anti-tumour effects in multiple melanoma cell lines, although to a different extent, (ii) that cannabinoids mediate their effects via CB1R, TRPV1 and PPARα leading to mitochondrial, caspase-mediated cell death and (iii) that cannabinoids have no antagonistic effect when combined with modern targeted therapy in vivo.

It has been shown that cannabinoids are able to inhibit angiogenesis by down-regulation of the vascular endothelial growth factor (VEGF) pathway in cancer cells [37]. This results in a normalized tumour vasculature that is smaller and contains fewer vessels that appear more differentiated and less leaky. In contrast, cannabinoids are able to promote tumour growth in HPV-positive head and neck cancer cell lines [38]. It was also shown that cannabinoids inhibit adhesion, migration and invasiveness of glioma, lung, breast and cervical cancer cells in vitro [39,40,41,42]. Interestingly, there have also been reports of in vitro assays showing that a variety of different cell lines from different tumour types (lung cancer, squamous cell carcinoma, bladder carcinoma, glioblastoma, astrocytoma and kidney cancer) show increased proliferation through EGFR and ERK signalling when treated with THC [12]. Similar findings were described when breast cancer cell lines were used in vivo and in vitro, demonstrating increased cell proliferation and metastatic spread by THC exposure [13]. Since melanoma is the tumour with the highest mutational burden, and, therefore, the tumour with the highest genetic diversity, it might be no surprise that melanoma cells have a high likelihood to exhibit primary resistance against cannabinoid treatment [43].

For these reasons, we were first of all interested in whether cannabinoids would have a comparable pro-apoptotic effect in several different melanoma cell lines. The commonly used A375 non-metastatic horizontally grown melanoma cell line showed a concentration-dependent decrease in viability when different concentrations of THC were used. This is in line with previously published reports [30]. To test their potential therapeutic efficacy, we tested cannabinoids in different metastatic melanoma cell lines, and, as shown in SK-Mel-28, THC alone had no significant effect on cell viability, which is again in line with the literature [30]. Furthermore, CBD alone showed only moderate effects in these cell lines but when THC and CBD were combined, there was a concentration-dependent reduction in cell viability in all melanoma cell lines. Viability was affected in immune cells only at higher concentrations (monocytes and lymphocytes) and in biphasic behaviour in eosinophils. This is of biological importance since it has been shown that immune cells play a pivotal role in fighting cancer including the modulation of immune checkpoint inhibitor-directed response [24,44]. Interestingly, all three combinations tested could reduce cell viability in eosinophils, although in a bimodal way as partially shown previously [44].

Up to date, two subtypes of cannabinoid G-coupled receptors, CB_1,_ and CB_2_, could be identified and fully characterized [7,8,9]. It has been demonstrated that CB1 is mainly expressed in different regions of the brain and, to a lesser extent, also in cardiomyocytes, adipocytes, hepatocytes and other cells [45]. In contrast, the CB2 receptor is mainly expressed in all hematopoietic cells [46]. In our study, we could demonstrate that the effect of THC is mediated through CB1. This might be no surprise since melanoma cells are originally derived from the neural crest and CB1 is mainly expressed in the brain [47,48,49]. However, treatment with CBD seems to be independent of CB1 receptor signalling. Other targets of cannabinoids including the peroxisome proliferator-activated receptors (PPAR) α and PPARγ, transient receptor potential vanilloid-1 (TRPV1) channels, G protein-coupled receptor (GPR) 55 and GPR35 have been described in the literature [11]. Importantly, we demonstrated that THC, as well as CBD, mediates its apoptotic effect through TRPV1. Further, there are reports available showing that PPARα activation is associated with a pro-apoptotic function by promoting BCL2 degradation and activation of caspase-3 [50,51]. In addition to TRPV1, we could confirm that both substances mediate their pro-apoptotic effects through PPARα signalling. Notably, it has been shown that breast cancer patients that lacked PPARα expression had a significantly shorter overall survival [52]. In melanoma, PPARα activation has been proposed to reduce metastatic potential via the down-regulation of AKT [53].

Apoptosis can be mediated by many pathways, including caspases. Caspase-dependent pathways can be further divided into the extrinsic and the intrinsic activation part, whereas the extrinsic pathway is mainly activated by death ligand receptors including TRAIL, CD95, TNF*α* and others [54]. This leads to the recruitment of Fas-associated death domain (FADD) in combination with caspase-8, which in turn activates down-stream caspases and initiates apoptosis [55]. The intrinsic pathway is activated by stress signals that lead to the release of pro-apoptotic factors such as apoptosis-inducing factor (AIF), cytochrome c or Smac/DIABLO from the mitochondrial intermembrane space, thereby quickly initiating mitochondrial membrane depolarization [56]. When A2058 cells were treated with cannabinoids, we observed that mitochondrial depolarization was disrupted as early as 2 h post treatment for up to 24 h. Importantly, we did not see any reduction in cell count until 24 h of cannabinoid treatment. Similar findings were made with cannabidiol in acute lymphoblastic leukemia cells [57]. Due to the highly lipophilic nature of cannabinoids, it has been proposed that they might have a direct effect on the mitochondrial membrane rather than it being a receptor-mediated action [58]. Nevertheless, there have also been some reports—which are in line with our findings—that activation of TRPV1 or CB1 can lead to mitochondria-mediated cell death [59,60,61]. Recent publications have suggested that cannabinoids might mediate their apoptotic effects by cytochrome c release from the mitochondrial membrane and consecutive activation of autophagy in different cancer cell lines [62,63]. When autophagy has been investigated in cannabinoid-treated cells, similar effects have been observed [30]. Surprisingly, we were not able to confirm previous findings that autophagy is involved in cell death mediated by cannabinoids in metastatic melanoma cells, and this might be due to the fact that autophagy depends on the activation of PTEN and p53 [64]. However, when melanoma exhibits a metastatic phenotype, loss of function mutations in PTEN and tp53 are commonly seen, and these mutations are needed for a more invasive and aggressive phenotype [21]. On the other hand, it has been reported that cannabinoids mediate their apoptotic activity through caspase activation in many types of cancers [65]. In line with these reports, we demonstrated that cannabinoids can activate the intrinsic caspase pathway through mitochondrial disruption and caspase-3/7 activation. However, it has been recently shown that caspase-3/7 activation does not necessarily lead to increased apoptosis in melanoma, especially when melanoma cells exhibit a metastatic phenotype [66]. Importantly, we demonstrated that caspase activation in our metastatic melanoma cell line led to increased apoptosis in an autophagy-independent way.

Cancer patients tend to use cannabis alongside clinical therapy to relieve their symptoms or in the belief that it is a good alternative to commonly used therapies [67]. Acute side effects of acute cannabis intake include euphoria, continuous laughter and talkativeness, sedation, lethargy and intensification of ordinary sensory experiences as well as perceptual distortion [68]. However, for cancer patients, the long-term effects of cannabis use are more important. It has been shown that chronic cannabis consumption can lead to the development of psychosis [69]. This is of importance since it was suggested that lifetime use of cannabis increases the risk of depression as well as greater suicidal ideation [70]. Especially important for cancer patients is the fact that lifetime consumption seems to be a relevant risk factor for suicidal attempts, suggesting that patients receiving such a supporting therapy need continuous monitoring as well as psychological support [71]. This has to be kept in mind when patients take it alongside their clinical therapy [72]. In addition, a recent study suggests that cannabinoids taken together with a commonly used immune checkpoint inhibitor reduce the efficacy of the latter one [73].

Thus, this raises two important questions regarding cannabinoid intake: first, what would be the direct effect of cannabinoids on cancer growth in vivo, and second, does commonly used modern pharmacological therapy interfere with cannabinoid intake?

In our in vivo model, we demonstrated that the clinically used combination of CBD and THC significantly reduced tumour growth in *BRAF^V600E^* mutated metastatic highly malignant melanoma cells. In previous works, Blazquez et al. paved the way for further cannabinoid studies, showing that the synthetic cannabinoids JWH-133 and WIN-55,212–2 have anti-proliferative and anti-metastatic effects in mice bearing B16 murine melanoma cells [74]. Simmerman et al. could demonstrate that CBD monotherapy can prolong survival in mice with B16F10 melanoma cells although not as long as cisplatin [75]. Similar findings were made when THC monotherapy was used in murine HCmel12 cells but not in murine B16 melanoma cells [76]. Armstrong et al. could further show that CHL-1 melanoma cells were affected by treatment with THC or CBD in combination with THC in a xenograft mouse model [30]. In addition to all these findings, we tested in our in vivo model if the commonly used MEK inhibitor trametinib has an effect on the *BRAF^V600E^* mutated A2058 metastatic melanoma cells and if the combination of CBD and THC interferes with trametinib in vivo. We demonstrated that there was no significant difference between all therapeutic groups. This is remarkable since there have been reports suggesting that cannabinoids might mediate their apoptotic potential over activation of MEK/ERK [77,78]. As opposed to these findings, there have been reports that cannabinoids can inhibit the MAPK pathway similar to MEK inhibitors in colon cancer cell lines, suggesting that there are cell type-specific cannabinoid-induced effects [79].

## 5. Conclusions

In summary, these data highlight the potential for cannabinoids to induce melanoma cell apoptosis in vitro and to limit their growth in vivo. Hence, cannabinoids might be used as supportive therapy in combination with modern targeted therapy in patients with metastatic melanoma, since they promote cell death in a caspase-dependent, autophagy-independent way.

## Data Availability

The data that support the findings of this study are available from the corresponding author upon reasonable request.

## Supplementary Materials

The following supporting information can be downloaded at: https://www.mdpi.com/article/10.3390/biology12050706/s1, **Figure S1:** Effect of cannabinoid, vemurafenib, AM251, AMG9810 and chloroquine treatment on melanoma cell viability. **Figure S2:** GW6471 can increase cell viability in cannabinoid treated melanoma cells and PPARa is rarely mutated in melanoma. **Figure S3:** Impact of cannabinoids on cell depolarization and impact of different antagonists on cannabinoid-induced reduction in melanoma cell viability. **Figure S4:** Effect of Trametinib treatment on melanoma cell viability and in-vivo treatment group characteristics including tumour area, age, mass and S100 expression. **Table S1:** Used drugs, cell lines, primers and Miscellaneous items. **Table S2:** Cell lines listed with their phenotype and evolutionary key mutations according to Shain et al. [21].
