# Supplementary material for: Cannabinoids Reduce Melanoma Cell Viability and Do Not Interfere with Commonly Used Targeted Therapy in Metastatic Melanoma In Vivo and In Vitro
Source: Biology (Basel). 2023 May 12;12(5):706. doi: 10.3390/biology12050706 (PMC10215168; doi:10.3390/biology12050706)
Supplement: Supplementary file 1 [file biology-12-00706-s001.zip › biology-2388308-supplementary.pdf]

Supplementary Materials

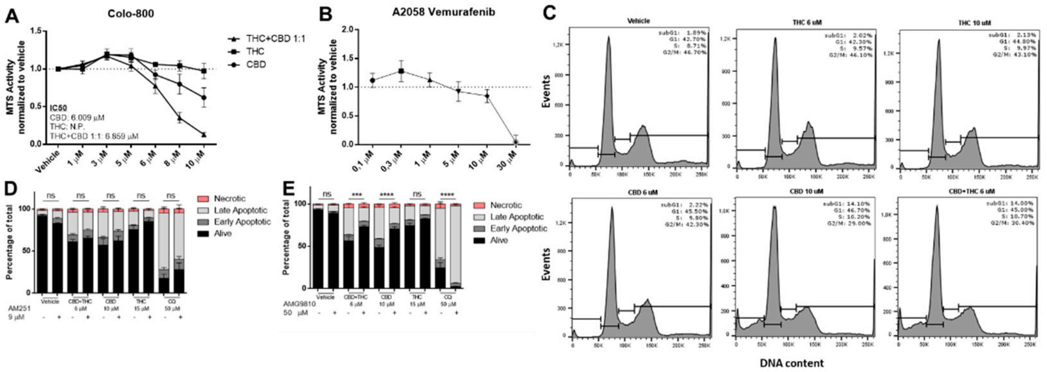

Figure S1. Effect of cannabinoid, vemurafenib, AM251, AMG9810 and chloroquine treatment on melanoma cell viability. (A) Colo-800 cells were treated with increasing dosage of cannabidiol (CBD), tetrahydrocannabinol (THC) or in a combination of both (1:1 ratio)(1, 3, 5, 6, 8 and 10 µM) for 24 h. **(B)** A2058 cells were treated with increasing dosages of vemurafenib (100 nM, 300 nM, 1 µM, 5 µM, 10 µM and 30 µM) for 24 h. **(C)** A2058 cells were treated for 24 h with vehicle, 6 µM of THC, 10 µM of THC, 6 µM of CBD, 10 µM of CBD or 6 µM of CBD and THC followed by cell cycle analysis using PE and FACS. Representative histograms of one experiment are shown. **(D-E)** A2058 cells were pre-treated with **(D)** 9 µM AM251 or **(E)** 50 µM AMG9810 followed by treatment with vehicle, 6 µM CBD and THC, 10 µM CBD, 15 µM THC or 50 µM chloroquine (CQ) for 24 h. All experiments have been performed in duplicates at least three times.

Data are presented as mean ± SD. * = p<0.05; ** = p<0.01; *** = p<0.001; **** = p<0.0001 and ns = non significant. IC50 = half maximal inhibitory concentration; N.P. = not possible.

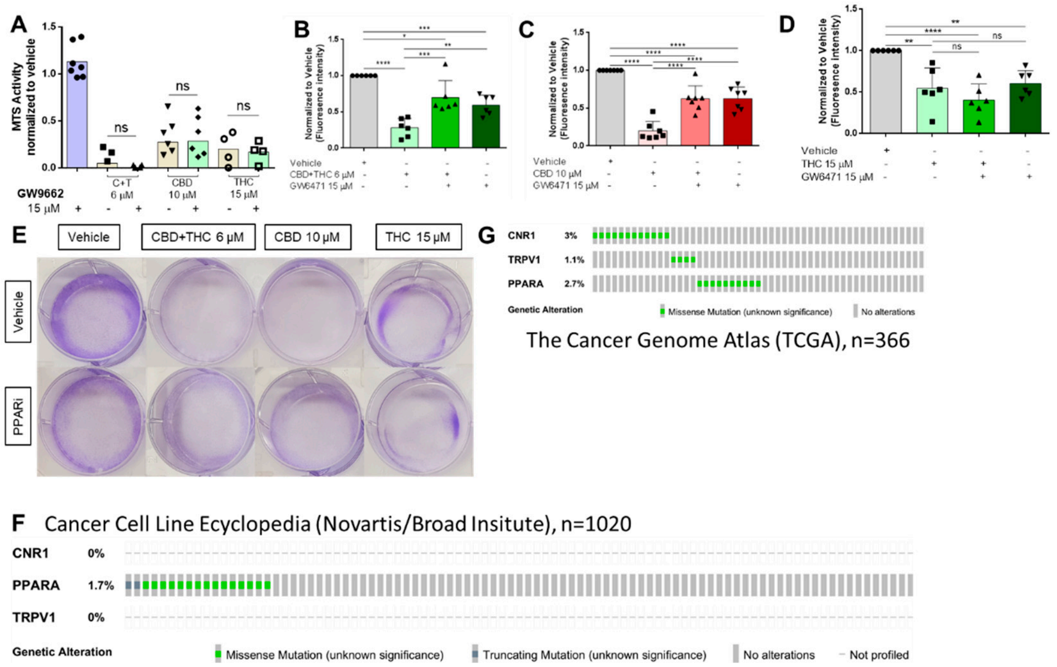

Figure S2. GW6471 can increase cell viability in cannabinoid treated melanoma cells and PPARa is rarely mutated in melanoma. (A) A2058 melanoma cells were treated with 15 µM of GW9662 (PPARγ antagonist) prior to treatment with 10 µM of CBD, 15 µM of THC or a 6 µM 1:1 combination of both for 24 h followed by cell viability assessment using the MTS assay (B-D) A2058 cells were treated with 15 µM of GW6471 (PPARα antagonist) prior to treatment with (B) a 6 µM 1:1 combination of CBD and THC, (C) 10 µM of CBD or (D) 15 µM of THC for 24 h. Afterwards cells were stained with crystal violet to visualize remaining attached cells and fluorescence intensity was measured by a spectrophotometer at 570 nm. (E) Representative image of A2058 cells stained with crystal violet after 24 h of the treatments indicated. (F-G) Mutations in CNR1, PPARα and TRPV1 were looked up using cbioportal.org (Accessed 19.11.2018) for (F) the Cancer Cell Line Encyclopaedia (Novartis/Broad Institute) and (G) melanoma patient samples from The Cancer Genome Atlas. Data are presented as mean ± SD. * = p<0.05; ** = p<0.01; *** = p<0.001; **** = p<0.0001 and ns = non significant.

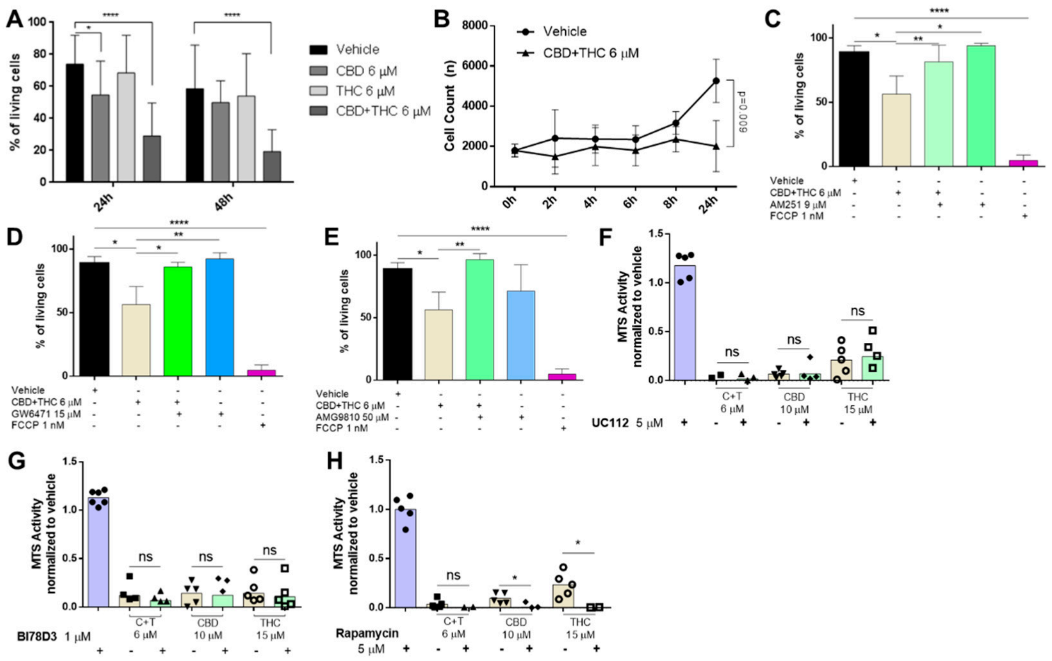

Figure S3. Impact of cannabinoids on cell depolarization and impact of different antagonists on cannabinoid-induced reduction in melanoma cell viability. (A) A2058 cells were treated with Cannabinoids (CBD and THC (6 µM)(C+T), CBD (6 µM) and THC (6 µM)) for 24 and 48 h and mitochondrial depolarization was investigated by flow cytometry. (B) A2058 cells were treated with Cannabinoids (CBD and THC (6 µM), CBD (6 µM) and THC (6 µM)) for 0, 2, 4, 6, 8 and 24 h and total number of living cells were counted by flow cytometry. (C-E) Prior to treatment with 6 µM of CBD and THC (ratio 1:1) for 24 h, A2058 cells were treated with (C) 9 µM AM251 (CB1 antagonist), (D) 15 µM GW6471 (PPARα antagonist) or (E) 50 µM AMG9810 (TRPV1 antagonist) followed by JC-1 staining and flow cytometric analysis. 1 nM of FCCP was used as a positive control. (F-H) Cells were treated with (F) 5 µM UC112 (IAP antagonist), with (G) 1 µM BI78D3 (JNK antagonist) or with (H) 5 µM rapamycin (mTOR antagonist) prior to treatment with 10 µM CBD, 15 µM THC or 6 µM a combination of CBD and THC (1:1) followed by MTS cell viability assessment. All experiments were performed in duplicates at least five times. Data are presented as mean ± SD. * = p<0.05; ** = p<0.01; *** = p<0.001; **** = p<0.0001 and ns = non significant.

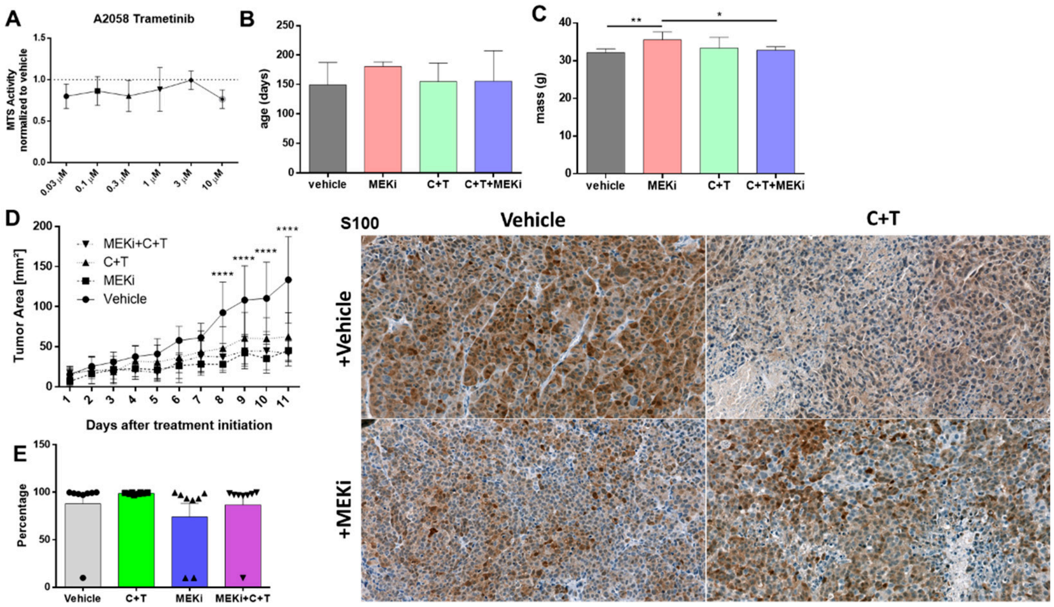

Figure S4. Effect of Trametinib treatment on melanoma cell viability and in-vivo treatment group characteristics including tumour area, age, mass and S100 expression. (A) A2058 cells were treated with increasing dosages (30 nM, 100 nM, 300 nM, 1 µM, 3 µM, 10 µM) of trametinib for 24 h and cell viability was assessed by MTS assay afterwards. (B-C) Mice age (B) and body mass (C) were recorded at the start of the treatment and compared between each group. (D) Tumour area was calculated every day from measured values until day 11 after treatment initiation. (E) Tumours were stained immunohistochemically for the melanoma marker S100.

Data are presented as mean ± SD. * = p<0.05; ** = p<0.01; *** = p<0.001; **** = p<0.0001 and ns = non significant.

Table S1. Used drugs, cell lines, primers and Miscellaneous items.

Drugs	Company (Location)	Cat. No.
AM251	Tocris (Abingdon, UK)	1117
AM630	Tocris (Abingdon, UK)	1120
AMG9810	Tocris (Abingdon, UK)	2316
UC112	Tocris (Abingdon, UK)	5251
BI78D3	Tocris (Abingdon, UK)	3314
AZ10417808	Tocris (Abingdon, UK)	2172
Z-VAD-FMK	Selleckchem (Houston, TX)	S7023
Rapamycin	Tocris (Abingdon, UK)	1292
GW6471	Tocris (Abingdon, UK)	4618
GW9662	Tocris (Abingdon, UK)	1508
Cannabidiol	Tocris (Abingdon, UK)	1570
Tetrahydrocannabinol	Gatt-koller (Absam, Austria)	609030009
Chloroquine	Tocris (Abingdon, UK)	4109
Bafilomycin A1	Tocris (Abingdon, UK)	1334
Vemurafenib	Selleckchem (Houston, TX)	S1267
Trametinib	Selleckchem (Houston, TX)	S2673
**Antibody**	**Company (Location)**	**Cat. No.**
PD-L1 Rabbit mAb (E1L3N)	Cell Signaling Technology Europe (Frankfurt am Main, Germany)	13684
LC3A/B Rabbit mAb (D3U4C)	Cell Signaling Technology Europe (Frankfurt am Main, Germany)	12741
Ki-67 Mouse mAb (8D5)	Cell Signaling Technology Europe (Frankfurt am Main, Germany)	9449
Cytochrome c Mouse mAb (6H2.B4)	Cell Signaling Technology Europe (Frankfurt am Main, Germany)	12963
Goat anti-Mouse IgG, Alexa Fluor 488	Thermo Scientific (Vienna, Austria)	A-11001
	**Miscellaneous**	
**Item**	**Company (Location)**	**Cat. No.**
Nunc™ Lab-Tek™ II Chamber Slide™ System	Thermo Scientific (Vienna, Austria)	154534
Caspase-Glo® 3/7 Assay Systems	Promega GmbH (Mannheim, Germany)	G8091
CellTiter 96® AQueous Non-Radioactive Cell Proliferation Assay (MTS)	Promega GmbH (Mannheim, Germany)	G5421
Penicillin-Streptomycin, 10,000 U/ml Penicillin, 10 mg/ml Streptomycin	PAN-Biotech GmbH (Aidenbach, Germany)	P06-07050
Trypsin 0.05 %/EDTA 0.02 % in PBS, w/o: Ca and Mg	PAN-Biotech GmbH (Aidenbach, Germany)	P10-023100
Dulbecco's Modified Eagle Medium (DMEM)	Thermo Scientific (Vienna, Austria)	41965039
DPBS, no calcium, no magnesium	Thermo Scientific (Vienna, Austria)	14190094
RPMI 1640 Medium	Thermo Scientific (Vienna, Austria)	21875034
Propidium iodide solution	Merck KGaA (Darmstadt, Germany)	P4864
Crystal Violet	Merck KGaA (Darmstadt, Germany)	C0775
Triton™ X-100	Merck KGaA (Darmstadt, Germany)	T8787
FITC Annexin V Apoptosis Detection Kit I	BD Bioscience (Schwechat, Austria)	556547
TRI Reagent®	Merck KGaA (Darmstadt, Germany)	93289
Fetal Bovine Serum (FBS)	Thermo Scientific (Vienna, Austria)	102701106
JC-1 Dye	Thermo Scientific (Vienna, Austria)	T3168
Histopaque®-1077	Merck KGaA (Darmstadt, Germany)	10771
4′,6-Diamidino-2-phenylindole dihydrochloride (DAPI)	Merck KGaA (Darmstadt, Germany)	D8417
Rhodamine Phalloidin	Thermo Scientific (Vienna, Austria)	R415
Eosinophil Isolation Kit, human	Miltenyi Biotec, Bergisch Gladbach, Germany	130-092-010
Kolliphor	Merck KGaA (Darmstadt, Germany)	C5135-500G

Cell lines		
**Name**	**Company/Institution**	**Cat. No./Donor**
A375	LGC Standards GmbH (Wesel, Germany)	CRL-1619
A2058	LGC Standards GmbH (Wesel, Germany)	CRL-11147
SK-Mel-28	LGC Standards GmbH (Wesel, Germany)	HTB-72
UACC-62	Research Institute of Molecular Pathology (IMP), Vienna Biocenter (VBC), Vienna, Austria	Dr. Anna Obenauf
Colo-800	Research Institute of Molecular Pathology (IMP), Vienna Biocenter (VBC), Vienna, Austria	Dr. Anna Obenauf
SK-Mel-30	Division of Oncology, Department of Internal Medicine, Medical University of Graz, Graz, Austria.	Prof. Martin Pichler
SBcl2	Biomedical Research, Medical University of Graz, Graz, Austria	Prof. Beate Rinner

Table S2. Cell lines listed with their phenotype and evolutionary key mutations according to Shain et al.[1].

**Cell line**	**Key mutations**	**COSMIC ID or source**
A375	$BRAF^{V600E}$	COSS906793
SBcl2	$NRAS^{Q61L}$, $CDKN2A^{del}$	Sini et al.[2]
SK-Mel-28	$BRAF^{V600E}$, APC^{S130G}, $PTEN^{T167A}$, $TP53^{L145R}$	COSS905954
UACC-62	$BRAF^{V600E}$, $PTEN^{P248fs*5}$	COSS905976
A2058	$BRAF^{V600E}$, $TP53^{V274F}$, $PTEN^{L112Q/V175fs*3}$, $MAP2K1^{P124S}$	COSS906792
Colo-800	$BRAF^{V600E}$, $TP53^{C135R}$	COSS906813
SK-Mel-30	$BRAF^{D287H/E275K}$, $NRAS^{Q61K}$, $TP53^{T284fs*21}$, $CDKN2A^{P114L}$, $APC^{G1339R/Q1406*}$	COSS909726

References

[1] A. H. Shain *et al.*, “The Genetic Evolution of Melanoma from Precursor Lesions,” *New England Journal of Medicine*, vol. 373, no. 20, pp. 1926–1936, 2015, doi: 10.1056/NEJMoa1502583.

[2] M. C. Sini *et al.*, “Genetic alterations in main candidate genes during melanoma progression,” *Oncotarget*, vol. 9, no. 9, Feb. 2018, doi: 10.18632/oncotarget.23989.
